# Dia-Interacting Protein (DIP) Imposes Migratory Plasticity in mDia2-Dependent Tumor Cells in Three-Dimensional Matrices

**DOI:** 10.1371/journal.pone.0045085

**Published:** 2012-09-14

**Authors:** Meghan M. Wyse, Jun Lei, Andrea L. Nestor-Kalinoski, Kathryn M. Eisenmann

**Affiliations:** 1 Department of Biochemistry, University of Toledo, Health Science Campus, Toledo, Ohio, United States of America; 2 Department of Surgery, University of Toledo, Health Science Campus, Toledo, Ohio, United States of America; NCMLS, Radboud University Nijmegen Medical Center, The Netherlands

## Abstract

Tumor cells rely upon membrane pliancy to escape primary lesions and invade secondary metastatic sites. This process relies upon localized assembly and disassembly cycles of F-actin that support and underlie the plasma membrane. Dynamic actin generates both spear-like and bleb structures respectively characterizing mesenchymal and amoeboid motility programs utilized by metastatic cells in three-dimensional matrices. The molecular mechanism and physiological trigger(s) driving membrane plasticity are poorly understood. mDia formins are F-actin assembly factors directing membrane pliancy in motile cells. mDia2 is functionally coupled with its binding partner DIP, regulating cortical actin and inducing membrane blebbing in amoeboid cells. Here we show that mDia2 and DIP co-tether to nascent blebs and this linkage is required for bleb formation. DIP controls mesenchymal/amoeboid cell interconvertability, while CXCL12 induces assembly of mDia2:DIP complexes to bleb cortices in 3D matrices. These results demonstrate how DIP-directed mDia2-dependent F-actin dynamics regulate morphological plasticity in motile cancer cells.

## Introduction

Metastatic cancer cells are capable of migration and invasion through extracellular matrix (ECM) barriers present in tissues, intravasation into lymphatics or bloodstream, extravasation, and dissemination and growth at a new site and these processes depend upon dynamic modulation of the cytoskeleton. Regulation of the actin cytoskeleton is fundamentally important for driving cell migration and invasion. Much work has focused upon the role of the Rho family of GTPase proteins in regulating cancer cell migration, invasion and metastasis. One family of GTPase effecter proteins that are critical for regulating the F-actin cytoskeleton during migration is the mammalian diaphanous-related (mDia) formins, including mDia1–3. mDia formins are evolutionarily conserved proteins that nucleate, elongate and, in some cases, bundle F-actin filaments that underlie cytoskeletal structures, such as filopodia, lamellipodia and ruffles [1], [2]. Like all mDia formins, mDia2 is maintained in an autoinhibited formation until a GTPase binds to the GTPase-binding domain [3]. GTPase binding to the formin releases the autoinhibited conformation, allowing protein effecters to bind.

mDia2 and other formin family members are implicated in the formation of actin-rich structures important for cellular motility in both normal and cancer cells (reviewed in [4]). Knockdown of the zebrafish mDia2 homologue zDia2 revealed a role in the formation of actin-rich protrusions controlling cellular migration during gastrulation [5]; zDia2 directed the formation of non-apoptotic membrane blebs in marginal deep cells at the germ-ring stage of gastrulation. Membrane blebbing is an initial indicator of cellular motility accompanying the transformation of non-motile blastomeres into motile blastula cells, suggesting a role for Dia2 homologues in cellular migration *in vivo*. mDia2 regulates Focal Adhesion (FA) stability in migrating epithelial cells [6], and was localized to the lamellar region and maintained a stable pool of F-actin critical to FA turnover. MDA-MB-231 breast cancer cell invasion was dependent upon mDia formins, and mDia2 was localized with Src to invadopodia; mDia2-depleted MDA-MB-231 cells had few invadopodia, pointing towards mDia2 as an important component in cancer cell invasion [7]. Deletion of the gene encoding murine mDia1, *DRF1,* revealed defects in assembly of the F-actin architecture important for T cell migration and trafficking [8], [9], in neutrophil polarization and chemotactic responses [10], [11], and in dendritic cell migration [12]. *In vitro*, formins control cortical actin contractility and their disruption promotes membrane blebbing and amoeboid morphology in cervical and prostate cancer cells [3], [13]. In the latter study, mDia2 depletion enhanced EGF-dependent blebbing and increased motility and invasion. Analysis of human prostate tumors revealed that 20% of primary and 60% of metastatic tumors had lost *DRF3/DIAPH3,* the gene encoding mDia2, linking mDia2 expression and/or function with metastasis; genomic loss of *DIAPH3* expression was subsequently linked to disease progression in invasive human breast and hepatocarcinoma, and siRNA-mediated suppression of *DIAPH3* enhanced prostate cancer lung metastasis in a tail vein injection xenograft model [14]. Other formins, (*i.e.,* mDia1, Formin-like 2, FHOD, FRL1/FMLN1) also drive membrane blebbing and amoeboid cancer cell motility *in vitro* [15], [16], [17], [18], [19], suggesting a conserved role for formins in regulating morphological plasticity during cancer cell motility. However, effecter proteins modifying formin-dependent signaling cues are poorly understood.

One mDia2 effecter protein is the diaphanous-interacting protein (DIP). DIP is a multi-domain protein implicated in stress fiber formation, cell migration, dendritic spine formation, vesicular trafficking and FA assembly [20], [21], [22], [23], [24]. DIP binds both mDia and WASp-family proteins, interacting through its amino-terminal SH3 domain with N-WASp or mDia via conserved proline-rich stretches within the FH1 domain [23], [25]. The mouse DIP orthologue, WISH, induced Arp2/3-mediated nucleation by interacting in a cdc42-independent manner with N-WASp [25]. DIP reportedly directly binds and activates the Arp2/3 complex, although this was not confirmed in cells [20]. The DIP knockout mouse showed slight diminution of actin accumulation in dendritic spines on explanted neurons [26], enhanced synaptic functions and motor function testing, and impaired MEF migration [27], [28].

A key understudied DIP feature is a conserved, leucine-rich repeat (LRR) region that directly binds mDia1 and mDia2 FH2 domains. We demonstrated that mDia2 binding to the DIP LRR is controlled by the Rho-governed autoregulatory mechanism mediated by Diaphanous-inhibitory domain (DID) and Diaphanous-autoregulatory domains (DAD) [3]. Full-length DIP and DIP LRR act as functional negative regulators of mDia2-mediated actin filament assembly and bundling, having no effect on mDia1-directed F-actin assembly. DIP inhibited mDia2-directed filopodia assembly and triggered non-apoptotic membrane blebbing, independent of either mDia1 or Arp2/3/WASp. These data identified a role for mDia2 in regulation of the cortical F-actin cytoskeleton and suggested that mDia2 interaction with DIP destabilizes the F-actin cortex to drive membrane blebbing, a hallmark of amoeboid motility. It remained uncertain whether the spatial and temporal association of mDia2 and DIP during membrane blebbing supported this mechanism, and whether DIP was required for driving amoeboid transitions in mesenchymal cancer cells.

Physiological stimuli driving amoeboid motility in cancer cells have yet to be identified. However, the chemokine CXCL12 (SDF-1α) induces membrane blebbing and amoeboid transitions in multiple experimental systems including zebrafish [29]. Zebrafish primordial germ cells bleb upon CXCL12 stimulation potentially through polarized activation of the CXCL12 receptor CXCR4. Upon CXCL12 stimulation, cells showed increased F-actin and cellular calcium [30], the latter demonstrated to be involved in membrane blebbing [29].

Here we examine the role of the mDia2:DIP axis in directing amoeboid transitions and driving blebbing in cancer cells. mDia2 and DIP co-localize to membrane blebs in constitutively blebbing M2 cells, and DIP depletion inhibits blebbing. We show a central role for expression and/or function of the mDia2:DIP axis in driving morphological plasticity in mesenchymal MDA-MB-231 and amoeboid MDA-MB-435S cells in both 2D and in 3D matrices. Finally, we identify CXCL12 as a driver of amoeboid transitions in MDA-MB-231 cells within 3D matrices, inducing spatial and temporal association of mDia2 and DIP. These data reveal an important role for DIP-directed mDia2 F-actin dynamics in regulating morphological plasticity in motile cancer cells.

## Results

### Spatial Localization of mDia2 and DIP in Constitutively Blebbing Cells

**Figure 1:**
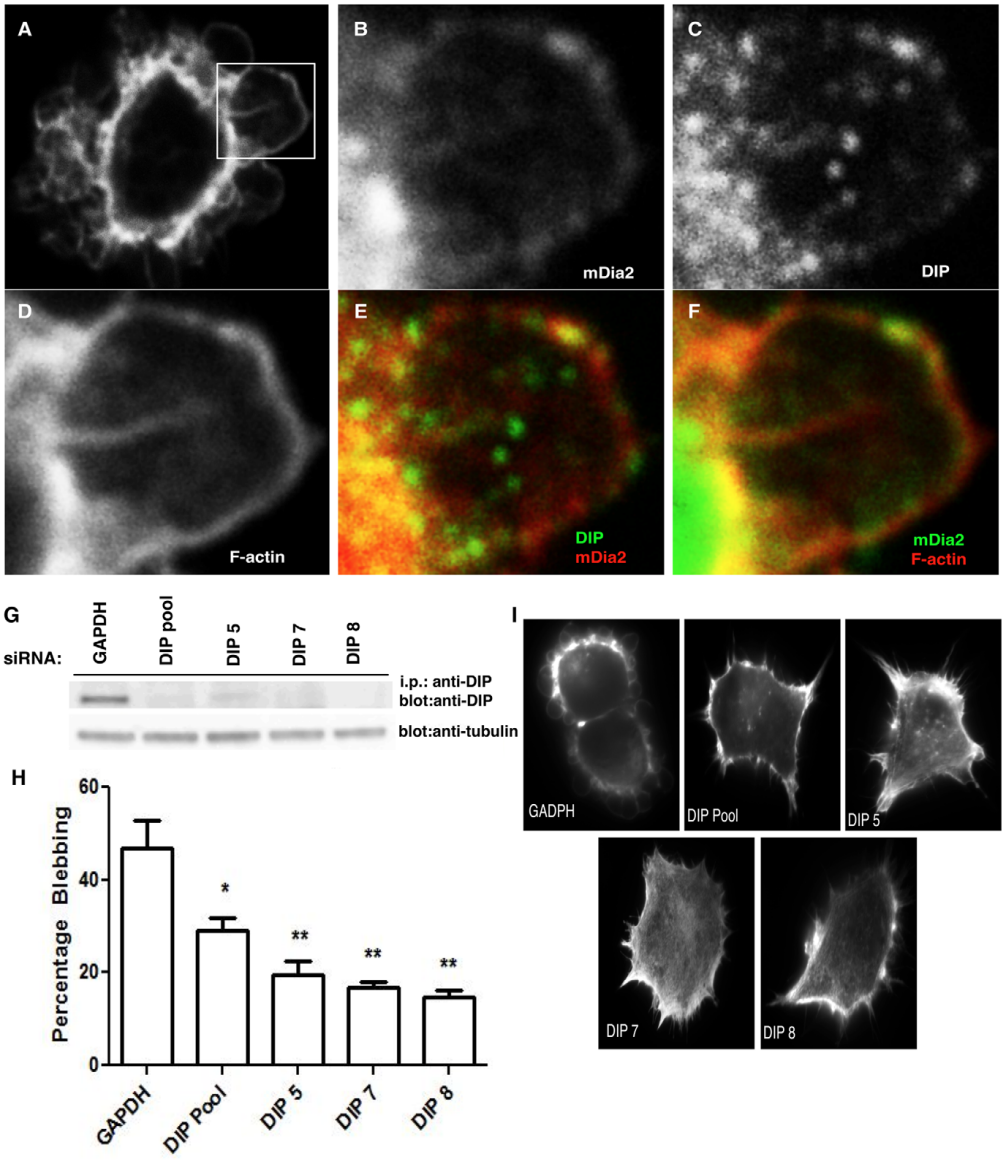
Requirement for DIP in non-apoptotic membrane blebbing. (A–F) Constitutively blebbing M2 melanoma cells were plated upon glass coverslips for 3 hrs, fixed, permeabilized and stained simultaneously with phalloidin and antibodies directed against mDia2 or DIP. Cells are visualized using a 63x objective with a confocal microscope. (G) DIP (pool or individual siRNAs) or GAPDH (control siRNA) depletion in M2 cells was confirmed 72 hrs post-transfection by i.p.-western blotting. Tubulin was used as a loading control. (H) Upon 72 hrs of siRNA-mediated DIP (pool or individual) or GAPDH depletion, blebbing M2 cells were quantified. The experiment was performed in triplicate with n>50 cells per condition. (I) M2 cells treated with GAPDH, DIP pooled or individual siRNAs for 72 hrs were plated upon glass coverslips, stained for phalloidin and were visualized using a 63x objective by confocal microscopy.

M2 melanoma cells have diminished expression of Filamin A, an F-actin cross-linking protein [31], [32], and constitutively extrude large blebs with extended half-lives. M2 cells were plated upon glass coverslips to stimulate profuse blebbing. The spatial localization of mDia2, DIP, and F-actin were assessed (Figure 1). mDia2 localized to large punctae at the bleb base and to the rim of expanded blebs (Figure 1B), where it co-localized with F-actin (Figure 1A, D, F). DIP localization was highly vesicular and concentrated primarily at the bleb bases where it co-localized with mDia2 (Figure 1C, E), implicating a potential role in bleb initiation and expansion. DIP also localized within the bleb and, to a lesser extent, to the rim of the bleb.

### DIP is Required for Non-apoptotic Plasma Membrane Blebbing

DIP suppression enhanced mDia2-directed filopodia assembly in HeLa cells, while DIP overexpression promoted blebbing [3]. To determine the requirement for DIP in blebbing, we depleted DIP from M2 cells using either pooled siRNA or individual siRNAs (or, as a control, GAPDH) (Figure 1G). Morphological evaluation revealed a dramatic shift towards an elongated, mesenchymal morphology devoid of blebs (Figure 1I). A decrease (p<0.001) in DIP-depleted blebbing cells was revealed relative to control cells (Figure 1H), indicating that DIP is a necessary component driving M2 blebbing and amoeboid morphology.

### Spatial and Temporal Regulation of DIP and mDia2

**Figure 2:**
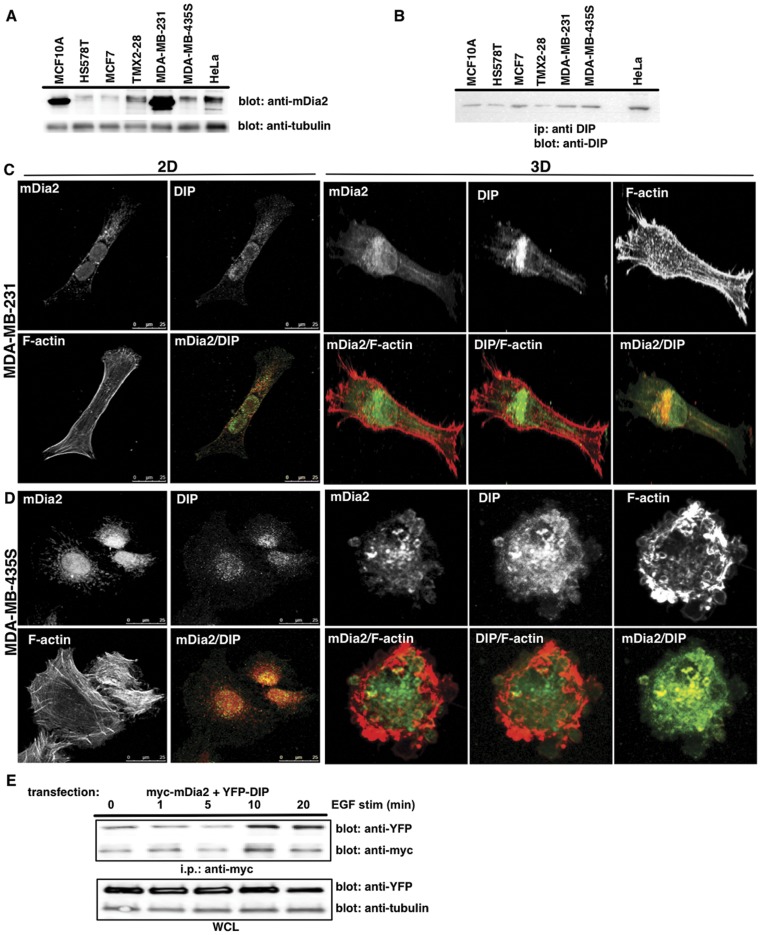
DIP and mDia2 expression in human breast cancer cells. mDia2 (A) and DIP (B) protein expression was assessed in a panel of human breast cancer cells by direct western or ip western blotting, respectively. HeLa lysates were used as a positive control known to express robust levels of both proteins. Tubulin is shown as a WCL loading control. (C, D) MDA-MB-435S or MDA-MB-231 cells were plated upon a thin-layer of 2D Type-I collagen (C) or within a thick layer of 3D Type-I collagen (D), and were incubated for 3 or 16 hrs, respectively. Endogenous mDia2, DIP or F-actin (phalloidin) are shown using a 40x objective with confocal microscopy. (E) MDA-MB-231 cells overexpressing myc-tagged mDia2 and YFP-DIP were stimulated with 10 nM EGF, and co-ips performed for mDia2-associated DIP. YFP and tubulin WCL blots are shown as loading controls.

M2 cells are not inherently motile; thus to study the effects of DIP upon morphological transitions in motile cancer cells, a panel of human breast cancer cell lines was screened for DIP and mDia2 expression, including epithelial- (MCF10A, MCF7), mesenchymal- (HS578T, MDA-MB-231) or amoeboid- (TMX2-28, MDA-MB-435S) shaped cells. The panel expressed varying mDia2 and DIP levels (Figure 2A and 2B), yet expression did not segregate with morphologies. MDA-MB-231 and MDA-MB-435S cells were utilized for the remainder of the studies as they are well characterized with morphological plasticity that can be experimentally manipulated [18], [33], [34]. We evaluated mDia2 and DIP localization in cells plated upon a thin 2D layer or within a thick 3D gel of type-I collagen (Figure 2C, D). In 2D, MDA-MB-435S cells (Figure 2D) were predominantly polarized with broad lamellae, mostly devoid of blebs. mDia2 expression was perinuclear with distinct tubular staining, whereas DIP staining was highly vesicular. MDA-MB-231 (Figure 2C) morphologies were mesenchymal, with perinuclear and tubular mDia2 staining and punctate DIP staining, which overlapped slightly in the perinuclear region.

In contrast, when embedded in 3D collagen matrices, MDA-MB-435S cells were predominantly amoeboid with hallmark blebs. mDia2 localized to blebs, and in particular, to bleb rims. DIP also localized to blebs, where it co-localized with mDia2. MDA-MB-231 cells were mesenchymal in 3D gels, with tubular mDia2 staining and associated perinuclear DIP.

We evaluated whether mDia2 and DIP complexed together upon EGF stimulation, which induced blebbing in HeLa [3] and in MDA-MB-231 cells (Figure S1). We transfected MDA-MB-231 cells with both mDia2 and DIP and stimulated with 10 nM EGF for 0–20 min. Upon 10 min of stimulation, DIP associated with mDia2 (Figure 2E), suggesting both a spatial and temporal regulation of the DIP and mDia2 complex in MDA-MB-231 and MDA-MB-435S cells.

### DIP Drives Morphological Plasticity in Migrating Cells in 3D Matrices

**Figure 3:**
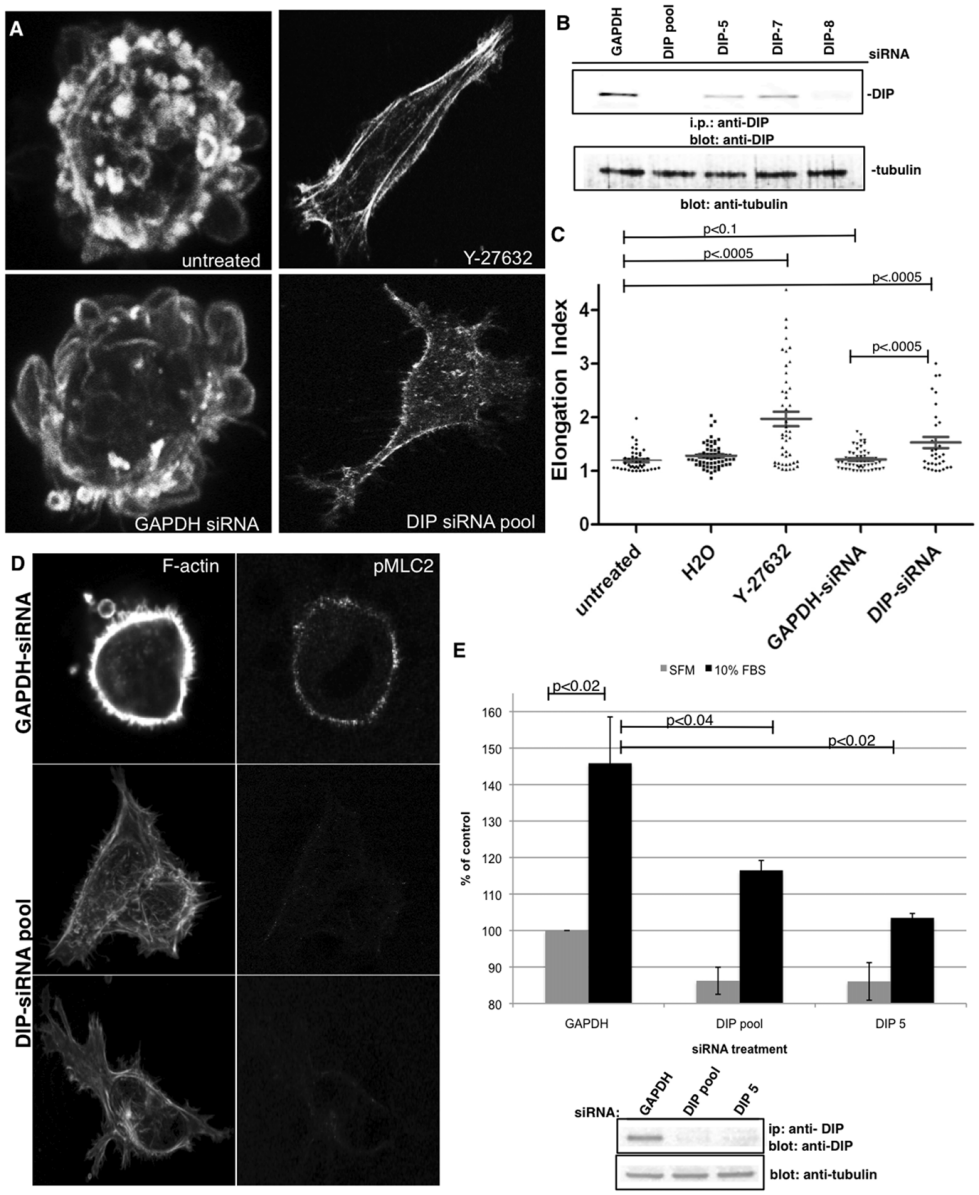
DIP directs amoeboid motility in breast cancer cells in 3D matrices. (A, B) MDA-MB-435S cells were treated with H_2_0 vehicle, 90 µM Rho-Kinase inhibitor Y-27632 or were depleted of DIP or control GAPDH via siRNA and were embedded into a thick layer of Type-I collagen and F-actin was visualized via phalloidin staining using a confocal microscope and a 40x oil objective. DIP knockdown was validated by i.p.-Western blotting, with tubulin shown as an i.p. input control blot. (C) Elongation indices were calculated for n>20 cells each condition, and experiments were repeated thrice. A representative experiment is shown. (D) MDA-MB-435S cells treated with either GAPDH or DIP siRNA for 72 hrs were embedded in collagen gels overnight, stained with phalloidin and anti-pMLC2 antibodies. (E) MDA-MB-435S cells treated with either GAPDH, DIP pool or DIP-5 siRNA for 72 hrs were allowed to invade for 48 hrs through transwells coated with collagen I gels. The extent of the knockdown at 120 hrs is shown by western blotting; tubulin is shown as an i.p. input control blot.

MDA-MB-435S cells were then treated with siRNA targeting GAPDH, DIP (Figure 3A–C) or treated with Rho kinase inhibitor Y-27632. Cells were embedded in collagen gels for 24 hrs, fixed and the F-actin architecture assessed. While untreated MDA-MB-435S cells retained a characteristic amoeboid and bleb-enriched morphology in 3D, Rho-kinase suppression drove a mesenchymal morphological switch, seen previously in these and other amoeboid tumor cells [16], [35], [36], [37]. DIP depletion revealed a mesenchymal transition, whereas GAPDH depletion yielded no transition. Elongation indices were calculated by dividing the long cellular axis by the short axis, revealing increases in MDA-MB-435S DIP-depleted cells relative to GAPDH (Figure 3C, p<0.0005). These data indicate a requirement for DIP for maintaining MDA-MB-435S amoeboid morphology in 3D matrices. Phospho-MLC2 levels were then assessed in DIP-depleted cells in 3D matrices (Figure 3D), as it is a hallmark characteristic of amoeboid morphologies. Indeed, control depleted amoeboid MDA-MB-435S revealed robust levels of pMLC2, while DIP-depleted, mesenchymal cells showed minimal pMLC2 levels, consistent with a loss of amoeboid morphology. Finally, the invasive capacity of DIP-depleted MDA-MB-435S cells was assessed using a transwell collagen invasion assay (Figure 3E). DIP-depleted cells were allowed to invade through collagen I for 48 hrs in response to a 10% serum gradient. Upon DIP depletion using either pooled or an individual siRNA and a concomitant mesenchymal transition, a marked reduction in invasion was observed relative to controls.

**Figure 4:**
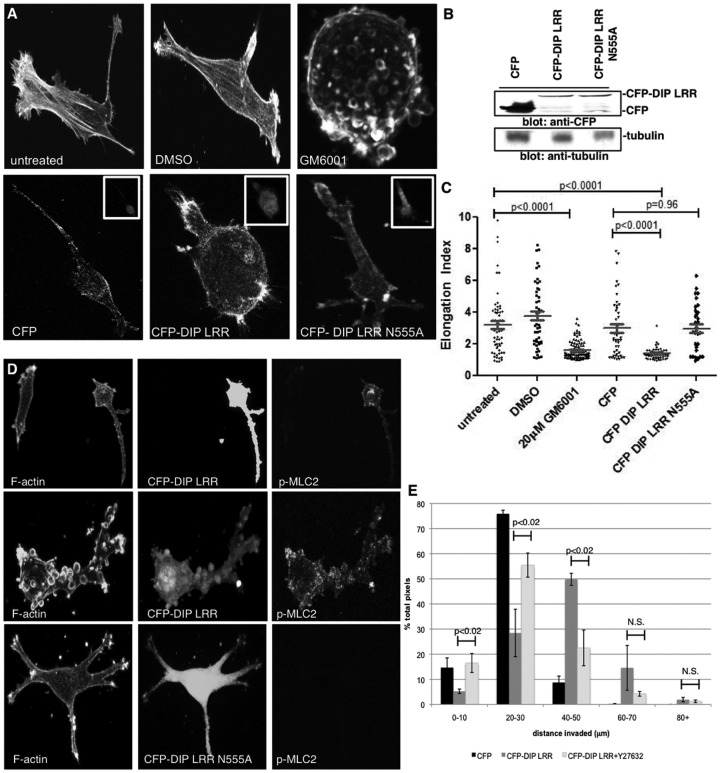
DIP LRR induces amoeboid motility in invading mesenchymal breast cancer cells. (A, B) MDA-MB-231 cells were treated with DMSO vehicle, 20 µm MMP-inhibitor GM6001 or were transfected with the indicated CFP-fusion proteins and were embedded in type-I collagen gels and stained as above. Insets validate the CFP-transfection. (C) Elongation indices were calculated as in 3C. (D) MDA-MB-231 cells transfected with either CFP-DIP LRR or CFP-DIP LRR N555A for 48 hrs were embedded in collagen gels overnight, stained with phalloidin and anti-pMLC2 antibodies. (E) MDA-MB-231 cells transfected for 48 hrs with CFP, CFP-DIP LRR or CFP-DIP LRR treated with 90 µM Y27632 reverse-invaded for 24 hrs through transwells coated with collagen I gels. Upon fixation, transwells were imaged by confocal microscopy. CFP pixels were calculated for 10 µm optical slices.

In contrast, MDA-MB-231 cells were treated with either DMSO, the MMP-inhibitor GM6001, or transfected with CFP or CFP-fused DIP LRR domains (Figure 4A, B). MMP inhibition induced an amoeboid transition in MDA-MB-231 cells [33], as did CFP-DIP LRR-expressing cells (Figure 4C, p<0.0001), relative to control CFP-expressing cells. CFP-DIP LRR-N555A, which abrogates binding to the mDia2 FH2 domain [3] and fails to disrupt FH2-dependent F-actin nucleation (K.M.E., unpublished observations), did not induce an amoeboid transition (Figure 4C, p = 0.96) suggesting that interaction between DIP and mDia2 is required for the DIP-driven amoeboid transition in MDA-MB-231 cells in 3D matrices. Furthermore, DIP LRR expression enhances pMLC2 levels in cells in 3D matrices, while DIP LRR N555A cells failed to do so (Figure 4D), confirming the amoeboid transition in DIP-LRR-expressing cells. Finally, invasion of amoeboid-transitioned CFP-DIP LRR-expressing cells was quantified by reverse invasion assay for 24 hrs (Figure 4E). Indeed, relative to CFP expression alone, expression of CFP-DIP LRR (and suppression of mDia2 FH2 activity) dramatically enhanced the proportion of cells migrating to depths beyond 30 µm; this enhanced invasion was sensitive to ROCK1/2 inhibition, as treatment with Y27632 diminished the proportion of cells invading beyond 30 µm. These data collectively indicate that the DIP-directed mDia2-dependent amoeboid transition impacts morphological plasticity in motile tumor cells.

### The Role of CXCL12 in Membrane Blebbing

**Figure 5:**
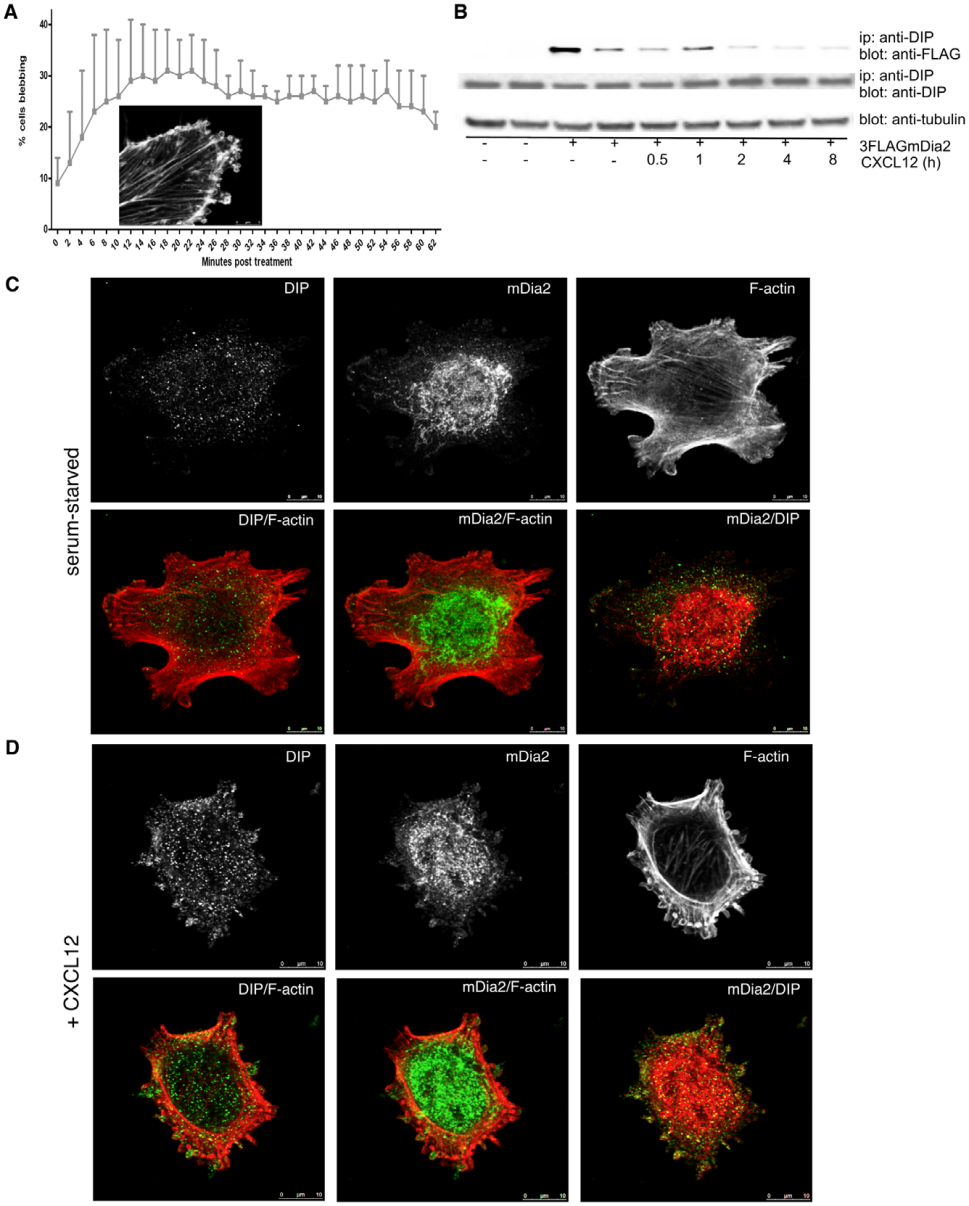
CXCL12 induces an mDia2:DIP complex and membrane blebbing. (A) MDA-MB-231 cells were plated upon glass coverslips and stimulated for the indicated times with 30 ng/ml CXCL12. Blebbing cells were enumerated in shown triplicate experiments where n>54. Inset: representative blebs in a fixed cell stained with phalloidin. (B) Cells transfected with the designated constructs were stimulated with 25 ng/ml CXCL12. Lysates were co-immunoprecipitated for DIP-associated mDia2 upon CXCL12 stimulation. Tubulin is shown as an i.p input loading control. (C, D) Serum-starved MDA-MB-231 cells were adhered to glass coverslips and stimulated for 30 min with 30 ng/ml CXCL12 before fixation. Endogenous DIP, mDia2 and the F-actin architecture are shown using a 63x oil objective.

The physiological stimuli driving amoeboid motility in cancer cells both *in vitro* and *in vivo* have yet to be identified; however, CXCL12 supports blebbing and amoeboid motility [29], [38] and CXCL12 is highly expressed in tissues that are prevalent sites of distant breast cancer metastasis [39], [40]. We examined if CXCL12 treatment induced blebbing. Expression of the receptor, CXCR4, was first confirmed in a panel of amoeboid and mesenchymal cells, with comparable expression across the panel (Figure S2). MDA-MB-231 cells were treated with CXCL12 (15–100 ng/ml) (Figure 5A and data not shown); 15–30 ng/ml stimulated profuse micro-blebbing (Figure 5A, inset) in 25–30% of cells within 15 min and was sustained through 60 min.

We assessed if CXCL12 induced a complex between DIP and mDia2, driving the DIP-mediated blebbing mechanism. MDA-MB-231 cells were transfected with FLAG-mDia2 and stimulated with CXCL12 for 0–8 hrs. Co-immunoprecipitation revealed that CXCL12 treatment increases the association between DIP and mDia2 through 1 hr, followed by sharp disassociation of the complex (Figure 5B). We then evaluated how CXCL12 treatment affected the spatial localization of DIP and mDia2. In untreated MDA-MB-231 cells, DIP localized to vesicular structures, while mDia2 was predominantly perinuclear and localized to tubular structures, with some perinuclear co-localization of proteins (Figure 5C). Upon CXCL12 treatment, copious F-actin-enriched small blebs formed with mDia2 and DIP co-localized to the blebs (Figure 5D), demonstrating that CXCL12 treatment induces the association of mDia2 with DIP to blebs.

**Figure 6:**
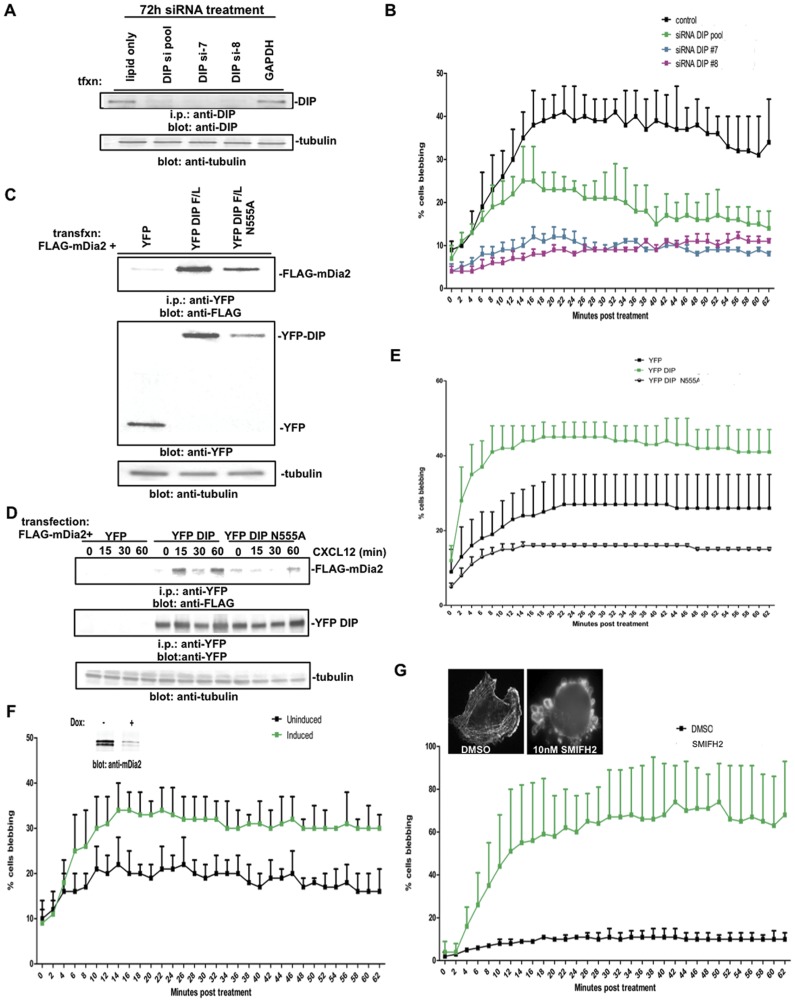
Suppression of the DIP:mDia2 axis impacts the CXCL12-driven blebbing mechanism. (A) MDA-MB-231 cells were transfected for 72 hrs with either GAPDH, DIP pool or individual DIP siRNAs and knockdown validated by i.p.-western blotting, with tubulin shown as a i.p. input control blot. (B) At 72 hrs post transfection, MDA-MB-231 cells were plated upon glass coverslips and stimulated for the indicated times with 30 ng/ml CXCL12 and blebbing cells enumerated, where n>100 cells. (C) HEK293T cells were transfected with 3XFLAG-mDia2 and either YFP, YFP-DIP or YFP-DIP N555A. Lysates were co-immunoprecipitated for DIP-associated mDia2. Tubulin is shown as a loading control for i.p. lysates. (D, E) MDA-MB-231 cells co-transfected for 48 hrs with 3XFLAG-mDia2 and YFP, YFP-DIP or YFP-DIP N555A were stimulated with 25 ng/ml CXCL12. Lysates were co-immunoprecipitated for DIP-associated mDia2 upon CXCL12 stimulation (D) and blebbing (E) was quantified as above. (F) MDA-MB-231 LN Luc cells stably expressing inducible miRNA directed against mDia2 were uninduced or induced with Doxycycline for 72 hrs and protein depletion validated (inset) by western blotting. (Un)induced cells were plated upon glass coverslips after 72 hrs induction and stimulated for the indicated times with 30 ng/ml CXCL12 and blebbing cells enumerated, where n>105 cells. (G) MDA-MB-231 cells were plated upon glass coverslips and, upon addition of either DMSO or 10 µm SMIFH2, blebbing was quantified in n>100 cells. Inset shows representative treated cells that were fixed and stained with phalloidin.

mDia2 or DIP were depleted from MDA-MB-231 cells to examine if DIP or mDia2 suppression affected the kinetics of CXCL12-induced blebbing. DIP depletion, via pooled or individual siRNAs, suppressed CXCL12-mediated blebbing, relative to controls (Figure 6A, B). To evaluate the contribution of the DIP LRR:mDia2 FH2 interaction upon morphological plasticity, YFP, YFP-DIP (full length) or YFP-DIP harboring an N555A point mutation was expressed and CXCL12-induced blebbing was assessed (Figure 6C-E). Despite the disruption in the LRR-FH2 interaction, DIP N555A robustly interacts with mDia2 in HEK293T cells, likely through the DIP SH3-mDia2 FH1 interaction [23]. Indeed, DIP and mDia2 co-expression in MDA-MB-231 cells revealed a robust CXCL12-inducible mDia2:DIP complex through 60 min (Figure 6D). Impairing the DIP LRR:FH2 interaction through the N555A mutation revealed delayed and diminished mDia2:DIP complex induction upon CXCL12 stimulation. Concurrently, a dramatic increase in CXCL12-induced blebbing was observed (p<0.0001 vs. YFP) upon DIP overexpression, while a modest, yet non-statistically significant (p>0.05 vs. YFP) increase was observed upon disruption of the DIP LRR-FH2 interaction via the N555A mutant (Figure 6E), indicating the importance of the mDia2 FH2:DIP LRR axis in driving morphological plasticity in response to CXCL12.

Furthermore, mDia2 depletion revealed both sustained and enhanced CXCL12-mediated blebbing (Figure 6F; p<0.01 relative to uninduced). Suppression of FH2 activity alone via treatment with the FH2 inhibitor SMIFH2, which targets both mDia1 and mDia2 FH2 activity [41], was sufficient to drive amoeboid transition and blebbing in the absence of CXCL12 (Figure 6G), consistent with findings in other tumor cells [41]. Interestingly, we demonstrated in *Drf1*-knockout MEFs that loss of mDia1 alone is not sufficient to drive blebbing in the presence or absence of EGF [3], indicating suppression of mDia2 and not mDia1 FH2 activity is critical for the blebbing mechanism. These data collectively reveal a role for DIP in the CXCL12-driven blebbing mechanism, through interaction with its binding partner mDia, therein acting as a functional negative regulator of FH2 activity; furthermore, suppression of mDia2 expression is sufficient to drive blebbing.

**Figure 7:**
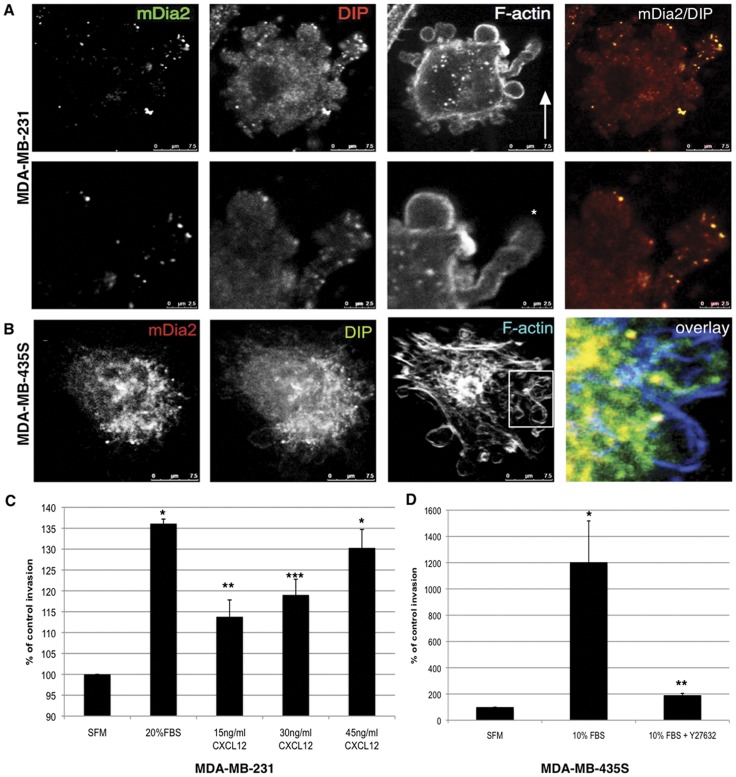
Colocalization of DIP and mDia2 to blebs in invading breast cancer cells. (A) MDA-MB-231 cells were subjected to a reverse invasion assay through Type-I collagen with 30 ng/ml CXCL12 in the upper well. After 24 hrs, cells were fixed and permeabilized. Endogenous DIP, mDia2 and the F-actin architecture are shown, and in the magnification of the compound bleb in A. (B) MDA-MB-435S cells were subjected to a reverse invasion assay through Type-I collagen with 10% serum in the upper well. After 24 hrs, cells were fixed and permeabilized. Endogenous DIP, mDia2 and the F-actin architecture are shown by confocal microscopy using a 40x oil objective. (C) MDA-MB-231 invasion towards increasing concentrations of CXCL12 was quantified after 48 hrs in a standard transwell collagen invasion assay. (D) MDA-MB-435S transwell collagen I invasion towards 10% serum in the presence or absence of 90µm Y27632 was quantified after 48 hrs. For C, * p<0.009; **p<0.05; ***p<0.025, relative to SFM. For D, *p<0.02; **p<0.009 relative to SFM.

Finally, we examined whether stimuli such as CXCL12 and FBS drive mDia2 and DIP to co-localize near/into the induced blebs in 3D matrices. MDA-MB-231 (Figure 7A, C) or MDA-MB-435S (Figure 7B, D) cells invaded through 3D matrices towards either CXCL12 or 10% FBS gradients, respectively. MDA-MB-231 cells are highly invasive and bleb in response to CXCL12, while amoeboid MDA-MB-435S cells are less migratory towards CXCL12, relative to FBS (data not shown). In contrast to the stellate MDA-MB-231 morphology in the untreated cells (Figure 5C), CXCL12 induced a dramatic amoeboid transition in invading cells, with cells enriched for large single and compound blebs (Figure 7A, C), and denoted by *), with punctate mDia2 and DIP co-localization to the blebs. MDA-MB-435S cells exposed to a 10% FBS gradient retained an amoeboid morphology with very large, exaggerated blebs (Figure 7B), and subsequent invasion through 3D collagen I matrices was sensitive to ROCK inhibition (Figure 7D). mDia2 and DIP were localized to large punctae at the bases of extruded blebs in MDA-MB-435S cells. Collectively, these data indicate that CXCL12 induces the spatial/temporal association of an mDia2 and DIP complex, therein driving DIP-dependent blebbing and amoeboid transitions in motile tumor cells in both 2D and 3D matrices.

## Discussion

Primary tumor cells continue to evolve novel modes of metastatic escape into distant tissues. These escape strategies require migratory plasticity, and are characterized by interconvertability between mesenchymal and amoeboid cancer cell motility programs. Morphological plasticity relies upon localized disassembly/reassembly of cytoskeletal structures that either drive and/or support the traction and protrusion cycles. Central to this process are Rho family GTP-binding proteins and their downstream effecters, formins. Here we demonstrate a functional role for mDia2 and its binding partner DIP in the interconversion of mesenchymal and amoeboid motility modes in cancer cells in 2D and 3D matrices. Co-recruitment of DIP and mDia2 to the plasma membrane is co-incident with bleb extrusion and blebbing is dependent upon DIP expression and mDia2 function. Our data reveal a physiological trigger for DIP-directed mDia2-dependent blebbing in mesenchymal breast cancer cells- CXCL12- which stimulates blebbing both in 2D and 3D matrices, while driving the assembly of the DIP:mDia2 complex to blebs.

The migratory plasticity in both 2D and 3D was dependent upon mDia2 and DIP expression/activation. As the Rho-dependent release of autoinhibited mDia2 is required for DIP:mDia2 interaction [3], these data suggest an important interplay between Rho activation and the DIP-driven mDia2-dependent amoeboid transition in both 2D and 3D matrices. This contrasts with 2D studies suggesting that DIP, through association with p190RhoGAP and VAV2, suppresses RhoA activity to negatively impact stress fiber formation and cellular migration [22]. Forced DIP overexpression in COS cells induced a transient decrease in GTP-RhoA upon EGF stimulation that was recoverable within 20 min. Rac-GTP levels were simultaneous elevated, consistent with lamellipodia formation, although DIP expression alone was not sufficient to activate Rac in another study [42]. In our study, in 2D and 3D matrices, EGF (Figure S1, data not shown) or CXCL12 induced an amoeboid transition in MDA-MB-231 cells through 60 min and DIP depletion in MDA-MB-435S cells induced a mesenchymal transition. These data suggest that Rho activity predominates the cellular signaling milieu to induce the formation of the mDia2:DIP signaling axis, thus maintaining an amoeboid morphology. However, it must be considered that spatial and temporal localization of DIP with its binding partners (*e.g*., mDia2, N-WASp, palladin, p53 IRES or Src [23], [25], [42], [43]) may control the assembly of DIP-directed complexes that functionally regulate actin dynamics. Importantly, our studies examine the contribution of DIP upon membrane dynamics in the context of 3D matrices, where the activity of Rho GTPases controlling migratory plasticity and the nature of actin-rich protrusion formed can be very distinct from those observed in 2D.

**Figure 8:**
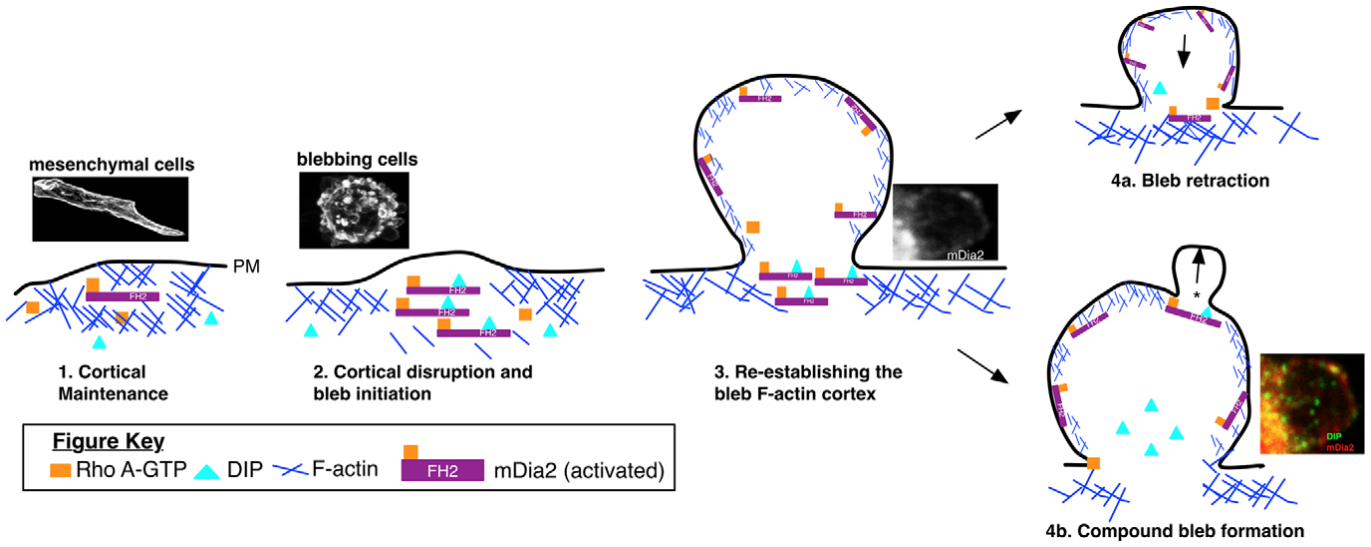
mDia2:DIP-driven mechanism for bleb and lobopodia formation: bulbous structures driving amoeboid motility in 3D matrices. 1. Cortical Maintenance: activated mDia2 maintains the F-actin cortex underlying the plasma membrane (PM); 2. Cortical disruption and bleb initiation: DIP binding to the mDia2 FH2 domain disrupts F-actin assembly and bundling, initiating cortical weakening and bleb expansion; 3. Re-establishing the bleb F-actin cortex: Upon bleb expansion, mDia2 enters the bleb and through interaction with activated Rho GTPases, re-establishes the F-actin cortex underlying the bleb; and 4a. Bleb retraction ensues, driven, in part, by ROCK and myosin-based contractility; 4b. Compound bleb formation: DIP binding the mDia2 FH2 domain at the rim of the bleb (noted as * in the inset) initiates a secondary break in the bleb cortex, leading to compound bleb, or lobopodia formation.

DIP and mDia2 predominantly co-associate at membranes associated with blebs in M2, MDA-MB-231 and MDA-MB-435S cells. These data point to a potential mechanism for blebbing and amoeboid transitions (Figure 8). In this model, localized GTP-Rho binds to and releases the autoinhibited mDia2 (Step 1), allowing it to complex with DIP at the cell cortex (Step 2). The consequence is disruption of F-actin assembly at the membrane and localized weakening of the actin cortex to induce bleb extrusion. Our data demonstrate distinct localization of mDia2 alone to the rim of extruded blebs (Figure 1B), revealing a second potential function for mDia2 in the re-assembly of the F-actin cortex preceding bleb retraction [44], [45], [46], [47] (Step 3, 4a). RhoA localized with F-actin to the rims of extruded and retracting blebs [47], suggesting a role for RhoA effecter proteins like mDia2 in maintaining blebs. However, mDia2 interacts with multiple Rho GTPases, including RhoA–C (reviewed in [48]) and Cdc42 [3], [49]. While it is uncertain if specific mDia2-interacting Rho GTPases drive the blebbing process, FHOD-mediated blebbing was dependent upon interaction with Rac [16], while RhoA was required for SH4-domain-induced blebbing [19]. Yet, GTP-Rac was shown to negatively regulate pMLC2, diminishing actinomyosin contractility and inhibiting the amoeboid motility program; Rho/ROCK pro-amoeboid signaling conversely directed Rac inhibition, via activation of the Rac GAP ARH-GAP22 [50], indicating a tightly controlled balance in Rho/Rac signaling in driving morphological transitions. Finally, we observed punctate DIP and mDia2 co-localization to the bleb rims and propose that this interaction induces a secondary break in the re-establishing F-actin cortex to mediate formation of compound blebs, or lobopodia [44], [51] (Step 4b).

Contrary to our previous biochemical studies [3] and the above mechanism, recent studies in *S. pombe* revealed that the yeast DIP orthologue, dip1p, failed to inhibit assembly of actin cable at the cell tips [52] and did not act as a formin regulator. However, the for3p FH2 domain shares less than 40% identity with the FH2 domain of its murine orthologue, mDia2, and subtle differences between mDia FH2 domains influence DIP’s ability to negatively regulate FH2-mediated F-actin assembly; despite their similarity, DIP binds both the mDia1 and mDia2 FH2 domains, yet only negatively regulates the latter [3]. As with mDia1, the for3p FH2 domain may preclude dip3p from acting as a negative regulator, whereas other *S. pombe* FH2 domain-containing formins may be potent targets for dip1p-mediated inhibition. Currently, the exact nature of subtleties in the primary sequence or secondary structures of FH2 domains conferring both the ability to bind and be regulated by DIP are unclear. Interestingly, while both the mDia1 and mDia2 FH2 domains polymerize F-actin, only mDia2 bundles F-actin filaments [53]. Further studies would be interesting to determine the parameters FH2 domains must conform to in order to be regulated by DIP.

It is uncertain if DIP-directed blebs in invading amoeboid cells are polarized towards a chemotactic gradient to drive protrusion and directional migration. Invading MDA-MB-231 cells formed copious blebs that polarized to the contracting cell rear proximal to the uropod, and migration speed and uropod formation were mDia dependent [7], [54], suggesting that blebbing may drive polarized contraction as opposed to forward membrane extension. However, Marshall and colleagues revealed the presence of a rigid, ezrin-enriched uropod at the rear of amoeboid, invasive A375 melanoma cells [55]; the presence of the rearward ROCK-dependent uropod inhibited proximal bleb formation, essentially establishing rear polarity through bleb inhibition. Our data show that MDA-MB-231 cells invading towards CXCL12 through collagen matrices have polarized compound blebs extending towards the gradient (Figure 7A), indicating a potential role for CXCL12 in directing bleb and/or cellular polarity. In fact, during zebrafish germline migration, CXCL12 gradients drive localized increases in Ca^2+^ to establish and stabilize protrusions that become the leading edge of the cell [29]. Experiments directed at understanding contributions of chemokine gradients to uropod formation and/or bleb polarization at the front/rear edge of migrating cancer cells are indeed warranted.

Finally, our data reveal a novel role for CXCL12 in inducing amoeboid transitions in MDA-MB-231 cells, and in recruiting the mDia2:DIP complex to blebs. CXCL12 and its receptor CXCR4 are implicated in the metastasis of several cancers including breast cancer metastasis to lung and lymph node [30], [56], [57]. CXCL12 is highly expressed in tissues that are prevalent sites of distant breast cancer metastasis [39], [40]. CXCR4 knockdown decreased MDA-MB-231 cell invasion [58], [59]. Therefore, amoeboid transitions in migrating breast cancer cells induced via exposure to CXCL12 may play a role in one or multiple steps in metastasis. Indeed, Rho degradation via the ubiquitin ligase Smurf1 locally impaired Rho/ROCK signaling and promoted mesenchymal motility, while Smurf1 inhibition induced amoeboid motility in tumor cells in subcutaneous xenografts [60], [61]. This transition was accompanied by dramatic increases in the number of tumor cells in and proximal to blood vessels, suggesting a linkage between amoeboid motility and intravasation. Ultimately, dynamic transition between mesenchymal and amoeboid motility may function during distinct stages of the metastatic process (*e.g.,* localized invasion) when the microenvironment requires morphological adaptation of tumor cells. Together, these studies and data presented herein demonstrate an important role for DIP-directed mDia2-dependent F-actin dynamics in regulating morphological plasticity in motile cancer cells, and reveal a novel role for CXCL12 in inducing amoeboid transitions within 3D matrices. These studies underscore the importance of understanding amoeboid motility in promoting mechanistic steps driving tumor metastasis.

## Methods

### Cell Culture, Transfection, and Time Lapse Image Acquisition

M2 (a kind gift from Dr. Maria Diakanova), HS578T, MCF7, MDA-MB-231, MDA-MB-435S, and HeLa were from ATCC; the MCF-7 (ATCC) ER-negative variant TMX2-28 (a kind gift from Dr. Kathleen Arcaro) was derived as previously described [62]; and MDA-MB-231 LN-Luc was from Caliper Life Sciences. Cells were maintained in DMEM (Gibco) supplemented with 10% FBS (v/v) and 100 units/ml penicillin, and 100 µg/ml streptomycin, while MCF10A (ATCC) were maintained as described [63] in a 37°C incubator with 5% CO_2_.

For plasmid transfections, MDA-MB-231 and M2 cells were transfected with either Lipofectamine LTX or Lipofectamine 2000 with Plus reagent, respectively (Invitrogen) per the manufacturer’s specifications. For transfection of siRNA in MDA-MB-435S cells, Dharmafect ON-TARGETplus SMARTpool (Thermoscientific) against human *NCKIPSD* (DIP or SPIN90), *DRF3* (mDia2) or GAPDH were used at 100 nM with Dharmafect 1 reagent. For miRNA infection, MDA-MB-231 LN-Luc cells were plated overnight and infected with viral particles at MOI 50. 1 µg/ml Doxycycline was used to induce expression for 72 hrs. Whole cell lysates were made using lysis buffer (0.5M Tris-HCl, pH 6.8, glycerol, 10% (w/v) SDS, 0.1% (w/v) bromophenol blue) supplemented with Dithiothreitol and heated for 10 min to 85°C. I.p. cell lysates were prepared in lysis buffer (20 mM Tris-HCl, pH 7.5, 100 mM NaCl, 1% NP40, 10% glycerol) containing 0.1M each sodium vanadate, aprotinin, pepstatin, leupeptin, dithiothreitol, and PMSF, and ips were performed as described [3]. Mouse anti-NCKIPSD (DIP) (Abcam), rabbit anti-mDia2 1358 (a kind gift from Dr. Art Alberts), rabbit anti-tubulin (Abcam), rabbit anti-myc (Santa Cruz), anti-FLAG (Sigma), or anti-GFP antibodies (Abcam) were used in analyses.

Time lapse imaging of M2 cells was acquired with an Olympus IX-81 (63x or 40x oil objective) with an environmental chamber that maintained cells at 37°C in a 5% CO_2_-containing atmosphere. Images were acquired at 10–30 sec intervals.

### Plasmids, siRNA and miRNA Sequences

Triple-FLAG tagged mDia2 was a kind gift from Brad Wallar and CFP-DIP LRR, myc-tagged mDia2 were previously described [3]. pSLIKneo-tet-On GFP-DIP, mDia2 and –GAPDH miRNA vectors were kind gifts from Dr Steve Matheson. The plasmid was derived using sequences (see Methods S1) subcloned into the pEM-TGmi-RC3 plasmid and recombined using the pSLIK-neo tet-On vector via the Gateway recombination system (Clontech). The vector was then cotransfected with pMD2.G and psPAX2 (Addgene) into HEK293T cells to produce lentiviral particles. siRNAs against human sequences were from Dharmacon as follows: DIP pool (cat. L-021376); individual siRNAs for DIP-5 (cat. J-021376-05), DIP-7 (cat. J-021376-07), DIP-8 (cat. J-021376-08); GAPDH pool (cat. D-001830).

### Immunofluorescence

Cells imaged in 2D were fixed with 4% paraformaldehyde in PBS (PFA), permeabilized with 0.2% Triton X-100 (TX100), washed, and incubated with the indicated primary antibody overnight at 4°C. Cells were incubated with Alexa-fluor labeled secondary antibody (Alexa 488, 546, or 647) and phalloidin (Molecular Probes). Coverslips were mounted onto glass slides with fluoromount G (Southern Biotech).

For 3D IF, cells were mixed with 2mg/ml Type I collagen (BD Biosciences), allowed to form a gel for 1 hr at 37°C and then incubated for 24 hrs in full growth medium. Cells were then fixed with 4% PFA, permeabilized with 0.2% TX100 and incubated with primary antibody overnight at 4°C. Cells were incubated with fluorescently labeled secondary antibody and phalloidin for 3 hrs at 37°C. Gels were covered with fluoromount G (Southern Biotech) and stored in the dark. Images were acquired using a TCS SP5 multiphoton laser scanning confocal microscope (Leica Microsystems) with laser lines of 458, 488, 514 and 633 in a sequential manner. Z stack images were acquired using 0.5 µm optical sections and creating a 3D reconstruction for image analysis using MetaMorph imaging software.

### Elongation Index

For calculation of elongation indices, cells were stained with Calcein AM FITC (Invitrogen), mixed into a 3D collagen matrix and gelled as above. Where indicated, control cells were treated with Y-27632 Rho-kinase inhibitor (Sigma) or MMP-inhibitor GM6001 (Sigma). 24 hrs post-embedding, cells were imaged by confocal microscopy with a 20x objective and 0.5 µm optical sections. Z-stacks were used to make projection images and Metamorph Image Analysis software was used to determine elongation index (cell long axis divided by cell short axis). Measurements were made for at least 20 cells per condition.

### Invasion Assays

Invasion assays were performed as described [64], casting 2.0 mg/ml collagen I gels upon Fluoroblock transwell inserts with 8 µm pores (BD Biosciences) using 50,000 cells per insert. 10%FBS was placed in the lower chamber to stimulate invasion. Cells were enumerated by staining inserts for 15 min with 0.5 µg/ml Calcein AM-FITC (Invitrogen) in PBS and enumerating using a fluorescent plate reader using 488ex:535em. Reversed invasion assays were performed as previously described with minor modifications [65]. Cells invaded through 2 mg/ml collagen I gels for 24 hrs, whereupon they were stained using antibodies directed against mDia2, DIP or with phalloidin. Cells were imaged by confocal microscopy using a 20x objective and 0.5 µm optical sections. Z-stacks were used to make 3D projections. When quantifying invasion, optical sections of 10 µm were analyzed for pixel intensities per slice. A 0 µm distance invaded was assigned based upon the appearance of both CFP pixels within the optical slice greater than the assigned background, and the appearance of pores as visualized by transmitted white light.

### Statistical Analysis

For blebbing quantification, the average percentage of blebbing cells for each sample was taken ± standard deviation. Elongation indices were calculated for at least 20 cells per sample and the average ± standard error was determined. Invasion was enumerated in triplicate and the average % of control (standard invasion) or % of total pixels (reversed invasion) was calculated. Data are expressed as average ±standard deviation.

A one-tail t-test was performed to evaluate statistical significance with a 95% confidence interval. A p value of 0.05 or less was considered statistically significant.

## Supporting Information

Figure S1 EGF stimulation induces blebbing in MDA-MB-231 cells. (Related to Figure 2) MDA-MB-231 cells were plated upon glass coverslips and incubated with serum-free medium overnight. Cells were left unstimulated or stimulated with 10 nM EGF for the indicated times, at which time the cells were fixed, stained with phalloidin (A) and blebbing cells enumerated (B). Data shown are an average of three experiments +/− standard deviations with n>100 cells.
(TIF)

Figure S2 CXCR4 receptor expression in amoeboid and mesenchymal cancer cell lines. (Related to Figure 5) Cell lysates were prepared from the designated cell lines and direct westerns performed using antibodies directed against CXCR4 (upper) or tubulin (lower), as a loading control.
(TIF)

Methods S1 Supporting Methods.
(DOCX)

## References

[1] Baarlink C, Brandt D, Grosse R (2010) SnapShot: Formins. Cell 142: 172, 172 e171. 10.1016/j.cell.2010.06.030 20603022

[2] Wallar BJ, Alberts AS (2003) The formins: active scaffolds that remodel the cytoskeleton. Trends Cell Biol 13: 435–446. 12888296 10.1016/s0962-8924(03)00153-3

[3] Eisenmann KM, Harris ES, Kitchen SM, Holman HA, Higgs HN, et al (2007) Dia-interacting protein modulates formin-mediated actin assembly at the cell cortex. Curr Biol 17: 579–591. 17398099 10.1016/j.cub.2007.03.024

[4] DeWard AD, Eisenmann KM, Matheson SF, Alberts AS (2010) The role of formins in human disease. Biochim Biophys Acta 1803: 226–233. 19941910 10.1016/j.bbamcr.2009.11.006

[5] Lai SL, Chan TH, Lin MJ, Huang WP, Lou SW, et al (2008) Diaphanous-related formin 2 and profilin I are required for gastrulation cell movements. PLoS One 3: e3439. 18941507 10.1371/journal.pone.0003439 PMC2565064

[6] Gupton SL, Eisenmann K, Alberts AS, Waterman-Storer CM (2007) mDia2 regulates actin and focal adhesion dynamics and organization in the lamella for efficient epithelial cell migration. J Cell Sci 120: 3475–3487. 17855386 10.1242/jcs.006049

[7] Lizarraga F, Poincloux R, Romao M, Montagnac G, Le Dez G, et al (2009) Diaphanous-related formins are required for invadopodia formation and invasion of breast tumor cells. Cancer Res 69: 2792–2800. 19276357 10.1158/0008-5472.CAN-08-3709

[8] Eisenmann KM, West RA, Hildebrand D, Kitchen SM, Peng J, et al (2007) T cell responses in mammalian diaphanous-related formin mDia1 knock-out mice. J Biol Chem 282: 25152–25158. 17595162 10.1074/jbc.M703243200

[9] Sakata D, Taniguchi H, Yasuda S, Adachi-Morishima A, Hamazaki Y, et al (2007) Impaired T lymphocyte trafficking in mice deficient in an actin-nucleating protein, mDia1. J Exp Med 204: 2031–2038. 17682067 10.1084/jem.20062647 PMC2118705

[10] Shi Y, Dong B, Miliotis H, Liu J, Alberts AS, et al (2009) Src kinase Hck association with the WASp and mDia1 cytoskeletal regulators promotes chemoattractant-induced Hck membrane targeting and activation in neutrophils. Biochem Cell Biol 87: 207–216. 19234535 10.1139/O08-130

[11] Shi Y, Zhang J, Mullin M, Dong B, Alberts AS, et al (2009) The mDial formin is required for neutrophil polarization, migration, and activation of the LARG/RhoA/ROCK signaling axis during chemotaxis. J Immunol 182: 3837–3845. 19265163 10.4049/jimmunol.0803838

[12] Tanizaki H, Egawa G, Inaba K, Honda T, Nakajima S, et al (2010) Rho-mDia1 pathway is required for adhesion, migration, and T-cell stimulation in dendritic cells. Blood 116: 5875–5884. 20881208 10.1182/blood-2010-01-264150

[13] Di Vizio D, Kim J, Hager MH, Morello M, Yang W, et al (2009) Oncosome formation in prostate cancer: association with a region of frequent chromosomal deletion in metastatic disease. Cancer Res 69: 5601–5609. 19549916 10.1158/0008-5472.CAN-08-3860 PMC2853876

[14] Hager MH, Morley S, Bielenberg DR, Gao S, Morello M, et al.. (2012) DIAPH3 governs the cellular transition to the amoeboid tumour phenotype. EMBO Mol Med. 10.1002/emmm.201200242 PMC3494074 22593025

[15] Han Y, Eppinger E, Schuster IG, Weigand LU, Liang X, et al (2009) Formin-like 1 (FMNL1) is regulated by N-terminal myristoylation and induces polarized membrane blebbing. J Biol Chem 284: 33409–33417. 19815554 10.1074/jbc.M109.060699 PMC2785185

[16] Hannemann S, Madrid R, Stastna J, Kitzing T, Gasteier J, et al (2008) The Diaphanous-related Formin FHOD1 associates with ROCK1 and promotes Src-dependent plasma membrane blebbing. J Biol Chem 283: 27891–27903. 18694941 10.1074/jbc.M801800200

[17] Kitzing TM, Sahadevan AS, Brandt DT, Knieling H, Hannemann S, et al (2007) Positive feedback between Dia1, LARG, and RhoA regulates cell morphology and invasion. Genes Dev 21: 1478–1483. 17575049 10.1101/gad.424807 PMC1891425

[18] Kitzing TM, Wang Y, Pertz O, Copeland JW, Grosse R (2010) Formin-like 2 drives amoeboid invasive cell motility downstream of RhoC. Oncogene 29: 2441–2448. 20101212 10.1038/onc.2009.515

[19] Tournaviti S, Hannemann S, Terjung S, Kitzing TM, Stegmayer C, et al (2007) SH4-domain-induced plasma membrane dynamization promotes bleb-associated cell motility. J Cell Sci 120: 3820–3829. 17959630 10.1242/jcs.011130

[20] Kim DJ, Kim SH, Lim CS, Choi KY, Park CS, et al (2006) Interaction of SPIN90 with the Arp2/3 complex mediates lamellipodia and actin comet tail formation. J Biol Chem 281: 617–625. 16253999 10.1074/jbc.M504450200

[21] Lee S, Lee K, Hwang S, Kim SH, Song WK, et al (2006) SPIN90/WISH interacts with PSD-95 and regulates dendritic spinogenesis via an N-WASP-independent mechanism. EMBO J 25: 4983–4995. 16990791 10.1038/sj.emboj.7601349 PMC1618117

[22] Meng W, Numazaki M, Takeuchi K, Uchibori Y, Ando-Akatsuka Y, et al (2004) DIP (mDia interacting protein) is a key molecule regulating Rho and Rac in a Src-dependent manner. EMBO J 23: 760–771. 14765113 10.1038/sj.emboj.7600095 PMC381003

[23] Satoh S, Tominaga T (2001) mDia-interacting protein acts downstream of Rho-mDia and modifies Src activation and stress fiber formation. J Biol Chem 276: 39290–39294. 11509578 10.1074/jbc.M107026200

[24] Zhu XL, Zeng YF, Guan J, Li YF, Deng YJ, et al (2011) FMNL2 is a positive regulator of cell motility and metastasis in colorectal carcinoma. J Pathol 224: 377–388. 21506128 10.1002/path.2871

[25] Fukuoka M, Suetsugu S, Miki H, Fukami K, Endo T, et al (2001) A novel neural Wiskott-Aldrich syndrome protein (N-WASP) binding protein, WISH, induces Arp2/3 complex activation independent of Cdc42. J Cell Biol 152: 471–482. 11157975 10.1083/jcb.152.3.471 PMC2196001

[26] Kim SM, Choi KY, Cho IH, Rhy JH, Kim SH, et al (2009) Regulation of dendritic spine morphology by SPIN90, a novel Shank binding partner. J Neurochem 109: 1106–1117. 19302483 10.1111/j.1471-4159.2009.06039.x

[27] Asrar S, Kaneko K, Takao K, Negandhi J, Matsui M, et al (2011) DIP/WISH deficiency enhances synaptic function and performance in the Barnes maze. Mol Brain 4: 39. 22018352 10.1186/1756-6606-4-39 PMC3208581

[28] Fukumi-Tominaga T, Mori Y, Matsuura A, Kaneko K, Matsui M, et al (2009) DIP/WISH-deficient mice reveal Dia- and N-WASP-interacting protein as a regulator of cytoskeletal dynamics in embryonic fibroblasts. Genes Cells 14: 1197–1207. 19778379 10.1111/j.1365-2443.2009.01345.x

[29] Blaser H, Reichman-Fried M, Castanon I, Dumstrei K, Marlow FL, et al (2006) Migration of zebrafish primordial germ cells: a role for myosin contraction and cytoplasmic flow. Dev Cell 11: 613–627. 17084355 10.1016/j.devcel.2006.09.023

[30] Holland JD, Kochetkova M, Akekawatchai C, Dottore M, Lopez A, et al (2006) Differential functional activation of chemokine receptor CXCR4 is mediated by G proteins in breast cancer cells. Cancer Res 66: 4117–4124. 16618732 10.1158/0008-5472.CAN-05-1631

[31] Cunningham CC, Leclerc N, Flanagan LA, Lu M, Janmey PA, et al (1997) Microtubule-associated protein 2c reorganizes both microtubules and microfilaments into distinct cytological structures in an actin-binding protein-280-deficient melanoma cell line. J Cell Biol 136: 845–857. 9049250 10.1083/jcb.136.4.845 PMC2132495

[32] Flanagan LA, Chou J, Falet H, Neujahr R, Hartwig JH, et al (2001) Filamin A, the Arp2/3 complex, and the morphology and function of cortical actin filaments in human melanoma cells. J Cell Biol 155: 511–517. 11706047 10.1083/jcb.200105148 PMC2198874

[33] Wolf K, Mazo I, Leung H, Engelke K, von Andrian UH, et al (2003) Compensation mechanism in tumor cell migration: mesenchymal-amoeboid transition after blocking of pericellular proteolysis. J Cell Biol 160: 267–277. 12527751 10.1083/jcb.200209006 PMC2172637

[34] Sahai E, Garcia-Medina R, Pouyssegur J, Vial E (2007) Smurf1 regulates tumor cell plasticity and motility through degradation of RhoA leading to localized inhibition of contractility. J Cell Biol 176: 35–42. Epub 2006 Dec 2026. 10.1083/jcb.200605135 PMC2063621 17190792

[35] Wilkinson S, Paterson HF, Marshall CJ (2005) Cdc42-MRCK and Rho-ROCK signalling cooperate in myosin phosphorylation and cell invasion. Nat Cell Biol 7: 255–261. 15723050 10.1038/ncb1230

[36] Demou ZN, Awad M, McKee T, Perentes JY, Wang X, et al (2005) Lack of telopeptides in fibrillar collagen I promotes the invasion of a metastatic breast tumor cell line. Cancer Res 65: 5674–5682. 15994941 10.1158/0008-5472.CAN-04-1682

[37] Sahai E, Marshall CJ (2003) Differing modes of tumour cell invasion have distinct requirements for Rho/ROCK signalling and extracellular proteolysis. Nat Cell Biol 5: 711–719. 12844144 10.1038/ncb1019

[38] Mizoguchi T, Verkade H, Heath JK, Kuroiwa A, Kikuchi Y (2008) Sdf1/Cxcr4 signaling controls the dorsal migration of endodermal cells during zebrafish gastrulation. Development 135: 2521–2529. 18579679 10.1242/dev.020107

[39] Hinton CV, Avraham S, Avraham HK (2010) Role of the CXCR4/CXCL12 signaling axis in breast cancer metastasis to the brain. Clin Exp Metastasis 27: 97–105. 18814042 10.1007/s10585-008-9210-2

[40] Muller A, Homey B, Soto H, Ge N, Catron D, et al (2001) Involvement of chemokine receptors in breast cancer metastasis. Nature 410: 50–56. 11242036 10.1038/35065016

[41] Rizvi SA, Neidt EM, Cui J, Feiger Z, Skau CT, et al (2009) Identification and characterization of a small molecule inhibitor of formin-mediated actin assembly. Chem Biol 16: 1158–1168. 19942139 10.1016/j.chembiol.2009.10.006 PMC2784894

[42] Teodorof C, Bae JI, Kim SM, Oh HJ, Kang YS, et al (2009) SPIN90-IRSp53 complex participates in Rac-induced membrane ruffling. Exp Cell Res 315: 2410–2419. 19460367 10.1016/j.yexcr.2009.05.010

[43] Ronty M, Taivainen A, Heiska L, Otey C, Ehler E, et al (2007) Palladin interacts with SH3 domains of SPIN90 and Src and is required for Src-induced cytoskeletal remodeling. Exp Cell Res 313: 2575–2585. 17537434 10.1016/j.yexcr.2007.04.030 PMC2000818

[44] Charras G, Paluch E (2008) Blebs lead the way: how to migrate without lamellipodia. Nat Rev Mol Cell Biol 9: 730–736. 18628785 10.1038/nrm2453

[45] Charras GT (2008) A short history of blebbing. J Microsc 231: 466–478. 18755002 10.1111/j.1365-2818.2008.02059.x

[46] Charras GT, Coughlin M, Mitchison TJ, Mahadevan L (2008) Life and times of a cellular bleb. Biophys J 94: 1836–1853. 17921219 10.1529/biophysj.107.113605 PMC2242777

[47] Charras GT, Hu CK, Coughlin M, Mitchison TJ (2006) Reassembly of contractile actin cortex in cell blebs. J Cell Biol 175: 477–490. 17088428 10.1083/jcb.200602085 PMC2064524

[48] Aspenstrom P (2010) Formin-binding proteins: modulators of formin-dependent actin polymerization. Biochim Biophys Acta 1803: 174–182. 19589360 10.1016/j.bbamcr.2009.06.002

[49] Peng J, Wallar BJ, Flanders A, Swiatek PJ, Alberts AS (2003) Disruption of the Diaphanous-related formin Drf1 gene encoding mDia1 reveals a role for Drf3 as an effector for Cdc42. Curr Biol 13: 534–545. 12676083 10.1016/s0960-9822(03)00170-2

[50] Sanz-Moreno V, Gadea G, Ahn J, Paterson H, Marra P, et al (2008) Rac activation and inactivation control plasticity of tumor cell movement. Cell 135: 510–523. 18984162 10.1016/j.cell.2008.09.043

[51] Kageyama T (1977) Motility and Locomotion of Embryonic Cells of the Medaka, Oryzias Latipes, during early development. Development, Growth and Differentiation 19: 103–110. 10.1111/j.1440-169X.1977.00103.x 37281789

[52] Basu R, Chang F (2011) Characterization of dip1p reveals a switch in Arp2/3-dependent actin assembly for fission yeast endocytosis. Curr Biol 21: 905–916. 21620704 10.1016/j.cub.2011.04.047 PMC3121306

[53] Harris ES, Rouiller I, Hanein D, Higgs HN (2006) Mechanistic differences in actin bundling activity of two mammalian formins, FRL1 and mDia2. J Biol Chem 281: 14383–14392. 16556604 10.1074/jbc.M510923200

[54] Poincloux R, Lizarraga F, Chavrier P (2009) Matrix invasion by tumour cells: a focus on MT1-MMP trafficking to invadopodia. J Cell Sci 122: 3015–3024. 19692588 10.1242/jcs.034561

[55] Lorentzen A, Bamber J, Sadok A, Elson-Schwab I, Marshall CJ (2011) An ezrin-rich, rigid uropod-like structure directs movement of amoeboid blebbing cells. J Cell Sci 124: 1256–1267. 21444753 10.1242/jcs.074849

[56] Andre F, Cabioglu N, Assi H, Sabourin JC, Delaloge S, et al (2006) Expression of chemokine receptors predicts the site of metastatic relapse in patients with axillary node positive primary breast cancer. Ann Oncol 17: 945–951. 16627550 10.1093/annonc/mdl053

[57] Kucia M, Reca R, Miekus K, Wanzeck J, Wojakowski W, et al (2005) Trafficking of normal stem cells and metastasis of cancer stem cells involve similar mechanisms: pivotal role of the SDF-1-CXCR4 axis. Stem Cells 23: 879–894. 15888687 10.1634/stemcells.2004-0342

[58] Liang Z, Yoon Y, Votaw J, Goodman MM, Williams L, et al (2005) Silencing of CXCR4 blocks breast cancer metastasis. Cancer Res 65: 967–971. 15705897 PMC3734941

[59] Lapteva N, Yang AG, Sanders DE, Strube RW, Chen SY (2005) CXCR4 knockdown by small interfering RNA abrogates breast tumor growth in vivo. Cancer Gene Ther 12: 84–89. 15472715 10.1038/sj.cgt.7700770

[60] Wang HR, Zhang Y, Ozdamar B, Ogunjimi AA, Alexandrova E, et al (2003) Regulation of cell polarity and protrusion formation by targeting RhoA for degradation. Science 302: 1775–1779. 14657501 10.1126/science.1090772

[61] Sahai E, Garcia-Medina R, Pouyssegur J, Vial E (2007) Smurf1 regulates tumor cell plasticity and motility through degradation of RhoA leading to localized inhibition of contractility. J Cell Biol 176: 35–42. 17190792 10.1083/jcb.200605135 PMC2063621

[62] Gozgit JM, Pentecost BT, Marconi SA, Otis CN, Wu C, et al (2006) Use of an aggressive MCF-7 cell line variant, TMX2–28, to study cell invasion in breast cancer. Mol Cancer Res 4: 905–913. 17189381 10.1158/1541-7786.MCR-06-0147

[63] Debnath J, Muthuswamy SK, Brugge JS (2003) Morphogenesis and oncogenesis of MCF-10A mammary epithelial acini grown in three-dimensional basement membrane cultures. Methods 30: 256–268. 12798140 10.1016/s1046-2023(03)00032-x

[64] Hooper S, Marshall JF, Sahai E (2006) Tumor cell migration in three dimensions. Methods Enzymol 406: 625–643. 16472693 10.1016/S0076-6879(06)06049-6

[65] Deakin NO, Turner CE (2011) Distinct roles for paxillin and Hic-5 in regulating breast cancer cell morphology, invasion, and metastasis. Mol Biol Cell 22: 327–341. 21148292 10.1091/mbc.e10-09-0790 PMC3031464

