# Supplementary figures and images for: Dia-Interacting Protein (DIP) Imposes Migratory Plasticity in mDia2-Dependent Tumor Cells in Three-Dimensional Matrices
Source: PLoS One. 2012 Sep 14;7(9):e45085. doi: 10.1371/journal.pone.0045085 (PMC3443221; doi:10.1371/journal.pone.0045085)

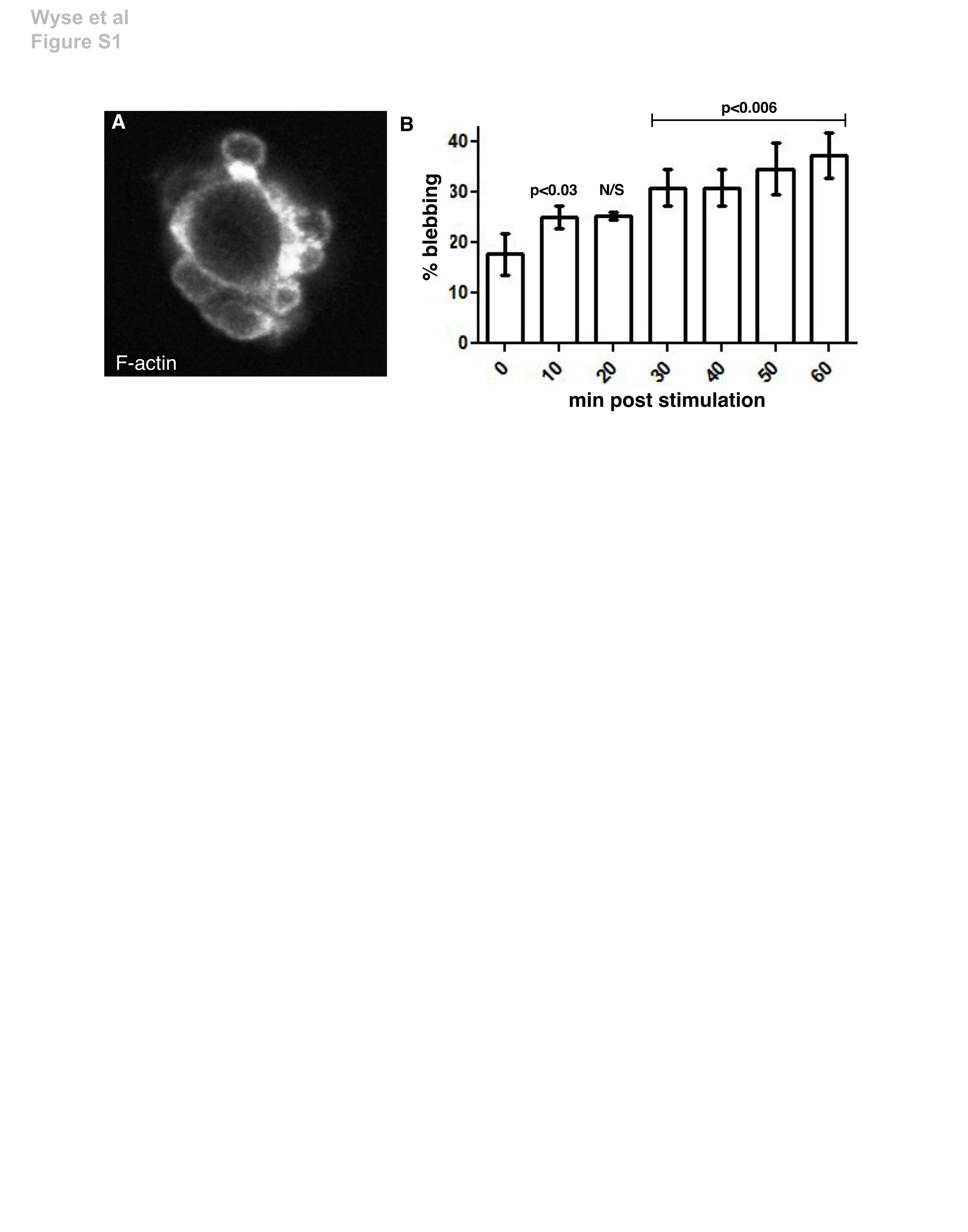

Supplement: Figure S1 — EGF stimulation induces blebbing in MDA-MB-231 cells. (Related to Figure 2) MDA-MB-231 cells were plated upon glass coverslips and incubated with serum-free medium overnight. Cells were left unstimulated or stimulated with 10 nM EGF for the indicated times, at which time the cells were fixed, stained with phalloidin (A) and blebbing cells enumerated (B). Data shown are an average of three experiments +/− standard deviations with n>100 cells. (TIF) [file pone.0045085.s001.tif]

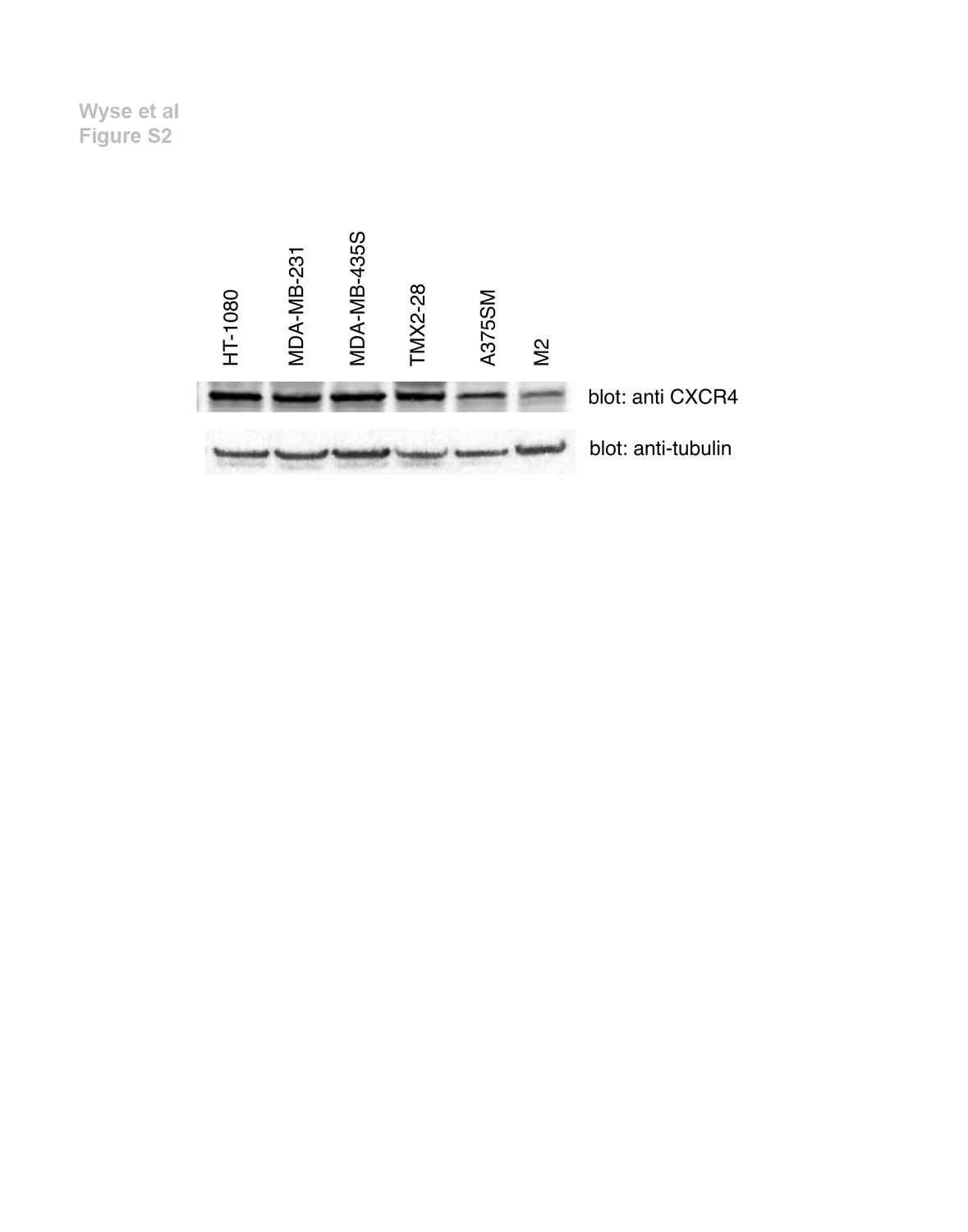

Supplement: Figure S2 — CXCR4 receptor expression in amoeboid and mesenchymal cancer cell lines. (Related to Figure 5) Cell lysates were prepared from the designated cell lines and direct westerns performed using antibodies directed against CXCR4 (upper) or tubulin (lower), as a loading control. (TIF) [file pone.0045085.s002.tif]
