# Supplementary material for: Dia-Interacting Protein (DIP) Imposes Migratory Plasticity in mDia2-Dependent Tumor Cells in Three-Dimensional Matrices
Source: PLoS One. 2012 Sep 14;7(9):e45085. doi: 10.1371/journal.pone.0045085 (PMC3443221; doi:10.1371/journal.pone.0045085)
Supplement: Methods S1 — Supporting Methods. (DOCX) [file pone.0045085.s003.docx]

**SUPPORTING METHODS**

**miRNA sequences for mDia2 and GAPDH**

GAPDH sequence-

5’AGCGAGCTCATTTCCTGGTATGACAATAGTGAAGCCACAGATGTATTGTCATACCAGGAAATGAGCG;

5’GGCACGCTCATTTCCTGGTATGACAATACATCTGTGGCTTCACTATTGTCATACCAGGAAATGAGCT

mDia2 sequence –

5’AGCGCCGCCAAGGAGCTTAATTATAATAGTGAAGCCACAGATGTATTATAATTAAGCTCCTTGGCGA;

5’GGCATCGCCAAGGAGCTTAATTATAATACATCTGTGGCTTCACTATTATAATTAAGCTCCTTGGCGG
